# Low back pain among professional bus drivers: a cross-sectional study from Bangladesh

**DOI:** 10.1186/s12889-023-16018-7

**Published:** 2023-06-19

**Authors:** Mohammad Hayatun Nabi, Mohammad Delwer Hossain Hawlader, Farah Naz, Saleka Raihana Siddiquea, Mehedi Hasan, Mosharop Hossian, Koustuv Dalal

**Affiliations:** 1 Department of Public Health, North South University, Dhaka, 1229 Bangladesh; 2 Department of Physiology, Green Life Medical College, Dhaka, 1205 Bangladesh; 3 Public Health Promotion and Development Society (PPDS), Dhaka, 1205 Bangladesh; 4 School of Health Sciences, Division of Public Health Science, Mid Sweden University, Sundsvall, Sweden

**Keywords:** Low back pain, Occupational health, Bus drivers, Quality of life, Bangladesh

## Abstract

**Background:**

Low back pain (LBP) is a common condition contributing to impaired quality of life among professional vehicle drivers. Our study aimed to assess LBP prevalence and associated factors among professional bus drivers in Bangladesh.

**Methods:**

A cross-sectional study was conducted among 368 professional bus drivers using a semi-structured questionnaire. A Nordic Musculoskeletal Questionnaire (NMQ) subscale was used to measure LBP. Multivariable logistic regression analysis was used to identify the factors associated with LBP.

**Results:**

In the last month, 127 (34.51%) participants reported experiencing pain or discomfort in the lower backside. Multivariable logistic regression analysis showed that age of more than 40 years (adjusted odds ratio (aOR): 2.07, 95% confidence interval (CI): 1.14 to 3.75), the income of more than 15,000 BDT per month (aOR: 1.91, 95% CI: 1.11 to 3.26), work duration more than ten years (aOR: 2.53, 95% CI: 1.12 to 5.70), working more than 15 days per month (aOR: 1.93, 95% CI: 1.02 to 3.65), working more than 10 h per day (aOR: 2.46, 95% CI: 1.05 to 5.75), poor condition of the driving seat (aOR: 1.80, 95% CI: 1.08 to 3.02), current smoking habit (aOR: 9.71, 95% CI: 1.25 to 75.15), illicit substances use (aOR: 1.97, 95% CI: 1.11 to 3.48), and four hours or less sleeping time per day (aOR: 1.83, 95% CI: 1.09 to 3.06) were positively associated with LBP.

**Conclusion:**

The high burden of LBP among the participants calls for a focus on this vulnerable group's occupational health and safety, with particular emphasis on implementing standard measures.

## Introduction

Low back pain (LBP) is a severe health condition that affects 577 million people worldwide [1]. It affects not only individuals who are immediately affected but the entire society. Existing literature suggests that as high as 80% of the adult population experienced low back pain at least once in their lifetime [2, 3]. Low back pain is a leading cause of the overall impact of musculoskeletal conditions, with 570 million cases worldwide and accounting for 7.4% of total years lived with disability globally [4]. People from lower socioeconomic status are more prone to experience LBP than those from higher [5]. LBP usually develops over time and can be intense, often leading to work absenteeism, work constraints, declining quality of life, and severe disability [6, 7].

Previous literature shows professional drivers are more prone to musculoskeletal disorders due to their job nature [8, 9]. Professional bus driving is an occupation that requires bus drivers to operate public transport over a more extended period than recommended working hours [10–12]. LBP has been reported most extensively among all occupational musculoskeletal disorders of professional drivers [13, 14]. A study conducted among USA and Sweden-based professional drivers found that nearly half of the Swedish bus drivers and about 80% of USA bus drivers reported experiencing LBP, which was much higher than the sedentary workers [15]. Bus drivers were ten times more likely to experience LBP than truck drivers, and 74.7% had LBP compared to 67.6% [16].

The possible contributing factors in developing occupational LBP among professional drivers include individual, occupational, and ergonomic factors. Individual factors such as age, short sleeping hours, tobacco smoking, and physical inactivity are possible risk factors for occupational LBP among professional drivers [17–19]. The most-reported occupational factors are daily working hours, working days per month, work duration, and work-related stress [16–19].

Concerning ergonomic factors, the drivers who complained more about uncomfortable seats, improper back support, insufficient workspace, and uncomfortable steering wheels experienced a significantly higher prevalence of LBP [17, 20, 21]. Furthermore, in Bangladesh, the situation of highways and the vehicle’s fitness might heighten the risk of developing LBP among intercity bus drivers. The road network gets defiled much quicker than the estimated durability because of inferior-quality bitumen and the vehicular roadworthiness evaluations need to be rigorously maintained [22]. Driving on roads plagued with potholes can lead to a tiresome experience, which can result in exposure to whole-body vibration (WBV) [16, 19, 23–25]. Studies have confirmed that WBV were higher on roads with speed bumps and lowered on smooth roads, irrespective of the type of Bus and driving seat used [16, 19, 23–25].

Even though bus drivers in Bangladesh are susceptible to occupational LBP the same way as drivers elsewhere, there is a considerable research gap regarding LBP among Bangladeshi intercity bus drivers. The current study aimed to fill the knowledge gap by estimating LBP prevalence among intercity bus drivers and assessing possible contributing factors. The findings will aid policymakers in prioritizing risk-reduction actions. Our efforts will also pave the way for upcoming studies to fill the research problems the current study should have covered.

## Methodology

### Study design and subjects

This cross-sectional study was conducted among conveniently selected intercity bus drivers from two purposively selected intercity bus terminals of Dhaka city (Dhaka North City Corporation and Dhaka South City Corporation). The required sample size for the study was 380 based on a 95% confidence interval (CI), the LBP prevalence among the bus drivers as 45% [21], and a 5% marginal error. The inclusion criteria were an age: of > 18 years; ≥ 1 year of bus driving experience [25–28]. The study excluded individuals with a history of operative or surgical procedures in the back and pelvic region, spinal surgery, abdominal surgery, injury to the back or spine caused by accidents, joint or bone disorder and deformities such as prolapse lumbar intervertebral disc, and chronic inflammatory conditions such as rheumatoid arthritis and ankylosing spondylitis. Twelve participants were excluded from the study due to incomplete responses, and finally, 368 respondents were considered for the analysis. The variables in the questionnaire were selected based on extensive literature review. Variables which were statistically significant in previous studies and have physiologic background were included [8, 16–21, 26]. However, during selection of the covariates, we emphasized on both statistical significance and physiologic background. Regarding statistical significance, we exercised flexibility in accepting variables with p value less than 0.10 [29].

### Dependent variable

The question on LBP condition was based on a subscale of the Nordic Musculoskeletal Questionnaire (NMQ) that measures discomfort or pain in the body's lower back, especially from the last rib to the buttock area, within the last 30 days before the study conducted [30]. For our current study, we utilized the Bangla version of the NMQ, which has been widely used in various studies [31–33] and proven to be valid and reliable among the Bangla-speaking population [33]. Participants were asked to rate the experience of LBP with two answering categories (“no pain or discomfort” and “experienced pain or discomfort”). Those who reported “no pain or discomfort” in the lower back were categorized as experiencing no LBP condition. Participants who answered otherwise were classified as having LBP.

### Independent variables

Data on sociodemographic, work-related, ergonomic, and lifestyle-related factors were collected using a semi-structured questionnaire. The sociodemographic factors included age, marital status, number of family members, crowding, monthly income, and years of education. In Bangladesh, bus drivers typically begin their careers at the age of 20, the minimum age required to obtain a professional driving license [34]. Section 28 of the Bangladesh Labour Act stipulates that an employee is required to retire upon reaching the age of 60 [35]. This age also corresponds with the broader societal definition of old age as ≥ 60 years [36]. Considering this information and the average working age for bus drivers, we have grouped our study participants accordingly. We have also categorized “Years of education” as “not educated” and “minimum literacy” and minimum literacy was defined as completing primary school level of education. In this study, participants were asked about their work-related factors like duration of working as a bus driver, monthly working days, and daily working hours. We also collected data on the condition of the buses and the participants' driving seats for the last three months. The term "satisfactory" for a bus driving seat refers to a driver's positive subjective assessment of its comfort and safety. In contrast, "poor" describes the opposite. When assessing a bus's overall condition in the past three months, "satisfactory" means the bus has operated without major issues, while "poor" reflects a negative assessment. The three-months timeframe was chosen for two reasons: 1) Maintenance schedules: Transportation companies typically maintain their buses every 3–4 months, so this period aligns with those schedules, giving a snapshot of the bus and seat condition [37, 38]. 2) Wear and tear: Buses and seats experience wear and tear over time. However, a three-month period might be enough to evaluate the driving seat condition by a driver without straying too far from the current state [39]. Questions on lifestyle-related factors like smoking habit, illicit substance use, betel leaf use (a type of leaf commonly consumed in Bangladesh with other ingredients.), sleeping hours and chronic diseases (Cancer, Diabetic Mellitus, Chronic Kidney Disease, Heart Disease) were also addressed in the study.

### Validation of the questionnaire

We used the Bengali version questionnaire for data collection. The English version questionnaire was developed first and then translated into Bengali and back-translated into English to ensure the content's quality and accuracy, which was reviewed by a panel of experts. Our expert panel, comprising one occupational health specialist, one injury prevention expert, one psychologist, and the other member was an experienced sociologist, guided the validation of our questionnaire. They confirmed its theoretical alignment with the broader low back pain construct and evaluated its face validity based on language clarity, relevance, and simplicity. A consensus was achieved through panel discussion. Content validity was also established via a two-stage process: computation of a content validity index for the item based on expert ratings, followed by a comprehensive review of the revised questionnaire. The questionnaire was also field-tested before the formal start of data collection.

### Data analysis

Stata version 15 for Mac (StataCorp., College Station, TX, USA) was used to analyze the study data. Descriptive statistics (frequencies and percentages) were presented for the categorical variables. Chi-square tests were performed to compare the categorical variables according to the experience of LBP. Multivariable logistic regression analysis modelled the statistical association between LBP and exposure variables. The model's independent variables were selected based on chi-squared p-values *(p* < *0.10)* [29]. The adjusted odds ratios (aORs) and corresponding 95% confidence intervals (CIs) were used to present the model's findings.

## Results

Three hundred sixty-eight male intercity bus drivers participated in the study. The average age of the participants was 41.14 ± 7.82 years (Table 1). The majority of the participants (97.28%) were married. In terms of family members, more than 60% of the participants (61.96%) reported having six or more members in their family. Nearly three-fourths (72.28%) of the participants had the lowest literacy, and about 60% of the bus drivers reported earning more than 15,000 BDT per month. From a crowding perspective, around eighty per cent (78.80%) of the participants lived in a room with ≤ 5 members. *p*-values from Chi-square tests indicate that age (*p* < 0.001) and monthly income (*p* < 0.001) have significant correlations with LBP.

**Table 1:** Sociodemographic characteristics of the study participants

**Variables**		**Low Back Pain**		***p*-value**
	**Total % Within Categories**	**Yes n (Row %)**	**No n (Row %)**	
**Age**				** < 0.001**
≤ 40 years	169 (45.92)	33 (19.53)	136 (80.47)	
≥ 41 years	199 (54.08)	94 (47.24)	105 (52.76)	
**Marital Status**				0.711
Married	358 (97.28)	123 (34.36)	235 (65.64)	
Unmarried	10 (2.72)	4 (40.00)	6 (60.00)	
**No. of family member**				0.330
≤ 5	140 (38.04)	44 (31.43)	96 (68.57)	
≥ 6	228 (61.96)	83 (36.40)	145 (63.60)	
**No. of person living in a room**				0.340
≤ 5	322 (87.50)	114 (35.40)	208 (64.60)	
≥ 6	46 (12.50)	13 (28.26)	33 (71.74)	
**Monthly Income (BDT)**				** < 0.001**
≤ 15,000	136 (36.96)	29 (21.32)	107 (78.68)	
≥ 15,001	232 (63.04)	98 (42.24)	134 (57.76)	
**Educational Status**				0.493
Not Educated	102 (27.72)	38 (37.25)	64 (62.75)	
Minimum Literacy	266 (72.28)	89 (33.46)	177 (66.54)	

*p*-value < 0.05 was considered statistically significant and marked as bold

The comparative picture of work-related factors of bus drivers with and without LBP is presented in Table 2. Bus drivers who were working for more than ten years reported high prevalence (41.16%) of LBP than those who (14.29%) were in the profession for ten years or less, and work duration was significantly associated with LBP (*p* < 0.001). Similar patterns were also observed for the number of working days per month (*p* = 0.001) and daily working hours (*p* = 0.001).

**Table 2:** Comparing the work-related variables between bus drivers with and without low back pain

**Variables**		**Low Back Pain**		***p*-value**
	**Total % Within Categories**	**Yes n (Row %)**	**No n (Row %)**	
**Work Duration (In years)**				** < 0.001**
≤ 10	91 (24.73)	13 (14.29)	78 (85.71)	
≥ 11	277 (75.27)	114 (41.16)	163 (58.84)	
**Working Days (Per month)**				**0.001**
≤ 15	89 (24.18)	18 (20.22)	71 (79.78)	
≥ 16	279 (75.82)	109 (39.07)	170 (60.93)	
**Daily Working Hours**				**0.001**
≤ 10	59 (16.03)	9 (15.25)	50 (84.75)	
≥ 11	309 (83.97)	118 (38.19)	191 (61.81)	

*p*-value < 0.05 was considered statistically significant and marked as bold

The ergonomic factors of the study participants, such as the bus condition, drove in the last three months, and the condition of the driving seat, are described in Table 3. Nevertheless, the data did not reveal any significant association between the LBP and ergonomic factors.

**Table 3:** Comparing the ergonomic variables between bus drivers with and without low back pain

**Variables**		**Low Back Pain**		***p*-value**
	**Total % Within Categories**	**Yes n (Row %)**	**No n (Row %)**	
**Condition of the Bus Drove in Last 3 months**				0.965
Satisfactory	153 (41.58)	53 (34.64)	100 (65.36)	
Poor	215 (58.42)	74 (34.42)	141 (65.58)	
**Condition of the Driving Seat of the Bus Drove in Last 3 months**				0.064
Satisfactory	235 (63.86)	73 (31.06)	162 (68.94)	
Poor	133 (36.14)	54 (40.60)	79 (59.40)	

The study participants were asked about lifestyle-related factors like smoking habits, illicit substance use, betel leaf use, sleeping hours, and chronic diseases. The findings are presented in Table 4. Nearly half of the illicit substance users (48.19%) complained about having significantly higher LBP than their counterparts (*p* = 0.003). A similar association was also found between smoking habits and LBP (*p* < 0.001). This study also reported a significant correlation between sleeping hours and LBP (*p* = 0.003).

**Table 4:** Comparison of lifestyle related factors of bus drivers with and without low back pain

**Variables**		**Low Back Pain**		***p*-value**
	**Total % Within Categories**	**Yes n (Row %)**	**No n (Row %)**	
**Smoking Habit**				** < 0.001**
Smokers	334 (90.76)	126 (37.72)	208 (62.28)	
Non-smokers	34 (9.24)	1 (2.94)	33 (97.06)	
**Illicit Substances**				**0.003**
Yes	83 (22.55)	40 (48.19)	43 (51.81)	
No	285 (77.45)	87 (30.53)	198 (69.47)	
**Betel Leaf Use (BL)**				0.067
Yes	170 (46.20)	67 (39.41)	103 (60.59)	
No	198 (53.80)	60 (30.30)	138 (69.70)	
**Sleeping Hours**				**0.003**
≤ 4	201 (54.62)	83 (41.29)	118 (58.71)	
≥ 5	167 (45.38)	44 (26.35)	123 (73.65)	
**Chronic Disease (s)**				0.116
Yes	60 (16.30)	26 (43.33)	34 (56.67)	
No	308 (83.70)	101 (32.79)	207 (67.21)	

*p*-value < 0.05 was considered statistically significant and marked as bold

The associated factors of LBP among the intercity bus drivers predicted by the multivariable logistic regression model are presented in Table 5. The results from the logistic regression model showed that after adjusting for confounders, age of more than 40 years (adjusted odds ratio (aOR): 2.07, 95% confidence interval (CI): 1.14 to 3.75), the income of more than 15,000 BDT per month (aOR: 1.91, 95% CI: 1.11 to 3.26), work duration more than ten years (aOR: 2.53, 95% CI: 1.12 to 5.70), working more than 15 days per month (aOR: 1.93, 95% CI: 1.02 to 3.65), working more than 10 h per day (aOR: 2.46, 95% CI: 1.05 to 5.75), poor condition of the driving seat (aOR: 1.80, 95% CI: 1.08 to 3.02), current smoking habit (aOR: 9.71, 95% CI: 1.25 to 75.15), illicit substances use (aOR: 1.97, 95% CI: 1.11 to 3.48), and four hours or less sleeping time per day (aOR: 1.83, 95% CI: 1.09 to 3.06) were positively associated with LBP.

**Table 5:** Results from logistic regression analysis – factors associated with low back pain

Variables	Crude Odds Ratio (95% CI)	Adjusted Odds Ratio (95% CI)
**Age (In years)**		
≤ 40 years	Reference	Reference
≥ 41 years	**3.69 (2.30 – 5.91)**	**2.07 (1.14 – 3.75)**
**Monthly Income (BDT)**		
≤ 15,000	Reference	Reference
≥ 15,001	**2.70 (1.66 – 4.39)**	**1.91 (1.11 – 3.26)**
**Work Duration (In years)**		
≤ 10	Reference	Reference
≥ 11	**4.20 (2.23 – 7.91)**	**2.53 (1.12 – 5.70)**
**Working Days (Per month)**		
≤ 15	Reference	Reference
≥ 16	**2.53 (1.43 – 4.47)**	**1.93 (1.02 – 3.65)**
**Daily Working Hours**		
≤ 10	Reference	Reference
≥ 11	**3.43 (1.63 – 7.24)**	**2.46 (1.05 – 5.75)**
**Condition of the Driving Seat of the Bus Drove in Last 3 months**		
Satisfactory	Reference	Reference
Poor	1.52 (0.97 – 2.36)	**1.80 (1.08 – 3.02)**
**Smoking Habit**		
Non-smokers	Reference	Reference
Smokers	**19.99 (2.70 – 147.96)**	**9.71 (1.25 – 75.15)**
**Illicit Substances**		
No	Reference	Reference
Yes	**2.12 (1.29 – 3.49)**	**1.97 (1.11 – 3.48)**
**Betel Leaf**		
No	Reference	Reference
Yes	1.50 (0.97 – 2.30)	0.94 (0.57 – 1.54)
**Sleeping Hours**		
≥ 5	Reference	Reference
≤ 4	**1.97 (1.26 – 3.07)**	**1.83 (1.09 – 3.06)**

Variables with significant OR at *p*-value < 0.05 level were marked as bold

## Discussion

In Bangladesh, bus drivers typically begin their careers at the age of 20, often they continue working into their late 50 s. The age and monthly income of the participants were found to be statistically significant, and this study revealed a much higher prevalence of LBP among Bus drivers aged ≥ 41. A similar significant statistical association was reported in studies conducted in Ethiopia, Brazil, India, Tanzania, and Nigeria [16, 19, 23–25]. However, many researchers found no such association [21, 26, 27]. Degenerative changes in the intervertebral disc, muscles and ligaments supporting the spinal column occur due to ageing, which can contribute to musculoskeletal conditions such as LBP [23, 28]. Participants with a monthly income of ≥ 15,001 BDT were at higher odds of having LBP than those who earned less when adjusted for other confounding variables. This significant association might be the culmination of existing trends in work and lifestyle-related variables such as more excellent working hours per day and month, longer working duration, and fewer sleeping hours in Bangladeshi transport workers. Many breaches in the maintenance of proper vehicle rules and laws directed by the Bangladesh Motor Vehicle Ordinance prevail, which shape the poor pay of bus drivers and which, in turn, might instigate the trends [10].

When adjusted for confounding variables, working duration of ≥ 11 years, working days of ≥ 16 days per month, working hours of ≥ 11 h per day and poor condition of the driving seat were all influencers of LBP. There are controversial findings about driving duration significantly associated with LBP across the literature. The prevalence of longer driving years is accompanied by cumulative vehicle-induced vibration, a risk factor for developing LBP [40]. Several studies agreed with our findings that a longer driving duration or more significant driving experience has a positive influence on the development of developing LBP [41–43]. Studies with opinions varying from our results indicated otherwise [16, 44]. A study conducted in Tanzania explained that more significant driving experience was a protective factor because experienced drivers tend to follow proper guidelines that avoid LBP [23].

Sally A. Hakim and Amira Mohsen found that those driving for more than 8 h per day are at a much higher risk of developing LBP. Driving for prolonged hours exposes drivers to musculoskeletal discomfort due to awkward sitting posture, poor seat condition, long time sitting without any body movements, and increased exposure to vibration, as the authors mentioned in their study [17]. Having more on-duty days per month obviously increases the WBV and thus, having more than 16 workdays per month might be another of the influencer as suggested by our findings. Several studies observed such associations, including epidemiological studies on occupational LBP, which identified static work posture, prolonged sitting, and exposure to vibration as three of the most common causes of LBP [3, 8, 16, 42, 44, 45]. Poor driving seat condition was observed to ergonomically affect the driver and increase the odds of having LBP in logistic regression. In a study conducted on bus drivers in the USA, drivers who had to use uncomfortable seats were reported to be at an eight times higher risk of LBP. As reported by epidemiological studies, musculoskeletal problems such as LBP result from postural stress due to prolonged sitting on the poor driving seat without good lumber or back support [26, 41, 44].

Lifestyle variables such as Smoking Habits and Illicit Substance Use positively influenced LBP in regression analysis. Smoking has been established as a risk factor in LBP development across literature [46]. However, multiple studies found no significant statistical association in their analysis [17, 24, 25, 42, 45]. Smoking has been found to contribute to spinal degenerative changes, and decreased perfusion to spinal cord which further promote LBP [46]. However, an open secret illicit substance use occurs daily among Bangladeshi bus drivers, especially those driving long distances at night. There is a lack of reports on illicit substance use among bus drivers in Bangladesh as it is a matter of sensitivity but illicit substance has been shown to be associated with acceleration of ageing which contributes to LBP development [46]. Similar to our findings regarding association of sleeping hour studies have observed poor sleep quality positively affecting development of LBP although the exact causal relationship is yet to be discovered [46]. A significant association found between illicit substance use and the prevalence of LBP is one of the strengths of this study. The presence of chronic diseases is mentioned to influence the development of LBP in several studies. However, this study did not find any such associations. A similar lack of associations can be noticed in a study conducted in Ethiopia [19].

Our findings may provide policymakers insights to comprehend the factors contributing to development of LBP in bus drivers which can be used in problem analysis, choice and implementation of measure tailored to address specific risk factors, allocate resources to support these interventions, evaluation and assessment. Regulating retirement age, working shifts, daily and weekly working duration, maintaining proper workplace and social support can be done to reduce the risk. Introducing mandatory rests or breaks can be done to reduce the uninterrupted sitting and driving period. Improving driving seat conditions, regular surveillance of vehicle and seat condition along with ergonomic interventions such as adjustable seats or backrests to support drivers who already have LBP, regular health checkup can largely address the factors responsible. Lastly, educational and training programs to promote healthy habits and reduce the impact of substance use on the health of bus drivers. Low cost interventions such as regular health assessments, ergonomic training and retraining, adjustable seating, driver rest breaks, vehicle condition monitoring could be the solution in reducing the high burden of LBP among bus drivers in Bangladesh [46].

### Limitation of this study

As a cross-sectional study using self-reported data, this study has limitations such as recall bias and reporting bias (over-reporting about work-related variables). Whole-body vibration, psychosocial factors, and night shift driving are established risk factors across the literature, but we did not include them in this study. Limitations in concluding any casual relationships persisted due to the nature of the design of this study. Not meeting the target sample size of 380 is another one of the limitations of this study.

## Conclusion

We found a high burden of LBP among intercity bus drivers in Bangladesh. The high prevalence of LBP may result in significantly impaired standard of living for bus drivers and should be addressed on a priority basis. Multivariable logistic regression analysis showed that LBP is significant in age, monthly income, duration of working as a bus driver, monthly working days, daily working hours, condition of driving seat, and smoking habit. Moreover, our results add a new dimension to the literature exploring illicit substance use's impact on experiencing LBP. For LBP prevention, it is necessary to introduce appropriate low-cost interventions with particular emphasis on implementing International Labor Organization (ILO)-guided occupational health and safety standards.

## Data Availability

The data underlying the results presented in this study will be provided on reasonable request to Dr. Delwer H. Hawlader. Email: mohammad.hawlader@northsouth.edu.
